# Static and fatigue strength of a novel anatomically contoured implant compared to five current open-wedge high tibial osteotomy plates

**DOI:** 10.1186/s40634-017-0115-3

**Published:** 2017-12-08

**Authors:** Arnaud Diffo Kaze, Stefan Maas, James Belsey, Alexander Hoffmann, Dietrich Pape

**Affiliations:** 1 University of Luxembourg, Faculty of Science, Technology and Communication, 6, rue R. Coudenhove-Kalergi, L-1359 Luxembourg, Luxembourg; 2 Department of Orthopedic Surgery, Centre Hospitalier de Luxembourg, L-1460 Luxembourg, Luxembourg; 3 Sports Medicine Research Laboratory, Public Research Centre for Health, Luxembourg, Centre Médical de la Fondation Norbert Metz, 76, rue d’Eich, L-1460 Luxembourg, Luxembourg; 4 Cartilage Net of the Greater Region, 66421 Homburg/Saar, Germany; 5 Department of Sport, Exercise & Health, University of Winchester, Sparkford Road, Winchester, SO22 4NR Hampshire England

**Keywords:** High tibial osteotomy (HTO), Osteoarthritis, Activmotion- TomoFix, PEEKPower, ContourLock, iBalance, Permanent deformation, Correction angle, Biomechanics, Mechanical stiffness, Static strength, Fatigue strength

## Abstract

**Background:**

The purpose of the present study was to compare the mechanical static and fatigue strength of the size 2 osteotomy plate “Activmotion” with the following five other common implants for the treatment of medial knee joint osteoarthritis: the TomoFix small stature, the TomoFix standard, the Contour Lock, the iBalance and the second generation PEEKPower.

**Methods:**

Six fourth-generation tibial bone composites underwent a medial open-wedge high tibial osteotomy (HTO), according to standard techniques, using size 2 Activmotion osteotomy plates. All bone-implant constructs were subjected to static compression load to failure and load-controlled cyclic fatigue failure testing, according to a previously defined testing protocol. The mechanical stability was investigated by considering different criteria and parameters: maximum forces, the maximum number of loading cycles, stiffness, the permanent plastic deformation of the specimens during the cyclic fatigue tests, and the maximum displacement range in the hysteresis loops of the cyclic loading responses.

**Results:**

In each test, all bone-implant constructs with the size 2 Activmotion plate failed with a fracture of the lateral cortex, like with the other five previously tested implants. For the static compression tests the failure occurred in each tested implant above the physiological loading of slow walking (> 2400 N). The load at failure for the Activmotion group was the highest (8200 N). In terms of maximum load and number of cycles performed prior to failure, the size 2 Activmotion plate showed higher results than all the other tested implants except the ContourLock plate. The iBalance implant offered the highest stiffness (3.1 kN/mm) for static loading on the lateral side, while the size 2 Activmotion showed the highest stiffness (4.8 kN/mm) in cyclic loading.

**Conclusions:**

Overall, regarding all of the analysed strength parameters, the size 2 Activmotion plate provided equivalent or higher mechanical stability compared to the previously tested implant. Implants with a metaphyseal slope adapted to the tibia anatomy, and positioned more anteriorly on the proximal medial side of the tibia, should provide good mechanical stability.

## Background

Osteoarthritis is the most frequent joint disorder with a worldwide increase over the last decade (Floerkemeier et al., 2013). High tibial osteotomy (HTO) is the principal intervention used for the treatment of medial compartment gonarthrosis with varus malalignment in young and active patients (Amendola & Bonasia, 2010; Pape et al., 2004). The closed-wedge HTO lost importance, in comparison to open-wedge, due to disadvantages such as: the requirement of a concomitant fibular osteotomy or disarticulation of the proximal tibiofibular joint, the potential injury to the common peroneal nerve, and difficulties with fine-tuning the correction during the operation (Hinterwimmer et al., 2012; Maas et al., 2013; Smith et al., 2013). The maintenance of correction after open-wedge HTO primarily depends on factors associated with the surgical technique and the implants used (Brinkman et al., 2008; Lobenhoffer & Agneskirchner, 2003; Spahn et al., 2006; Spahn et al., 2007). Precise preoperative planning and high primary fixation stability of the implant are required for a good outcome (Pape et al., 2004). New implants for HTO, such as the size 2 Activmotion plate (Table 1, Group VI) of the company Newclip Technics (Haute*-*Goulaine, France), are continuously introduced into the market. They have different shapes and also have varying biomechanical and material properties. It is important to quantify and compare the stabilising effect of these implants. Diffo Kaze et al. performed a biomechanical study (Diffo Kaze, 2016; Diffo Kaze et al., 2015) that compared the following five implants: the TomoFix small stature (TomoFix sm) and TomoFix standard (TomoFix std) plates of Synthes Gmbh (Oberdorf, Switzerland), and the ContourLock plate, iBalance implant and second generation PEEKPower plate of Arthrex (Munich, Germany) (Table 1, Group I to V). All of the plates are precontoured to fit the medial proximal tibia. The iBalance implant is inserted centrally onto the medial surface inside the osteotomy gap. The size 2 Activmotion, which has a metaphyseal slope adapted to the tibia anatomy, is positioned onto the antero-medial surface of the tibia head while the other implants have their proximal part centred onto the medial surface of the tibia head. The purpose of the present study was to compare the mechanical static and fatigue strength of the size 2 osteotomy plate “Activmotion” with five other implants designed for the treatment of medial knee joint osteoarthritis, using a testing procedure that has already been defined, used and published (Diffo Kaze, 2016; Diffo Kaze et al., 2015; Maas et al., 2013). It was hypothesised that the new Activmotion plate (Table 1, Group VI), which is also made from titanium, and affixed onto the antero-medial side, should provide sufficient mechanical stability, comparable to the previously tested implants.

**Table 1:**
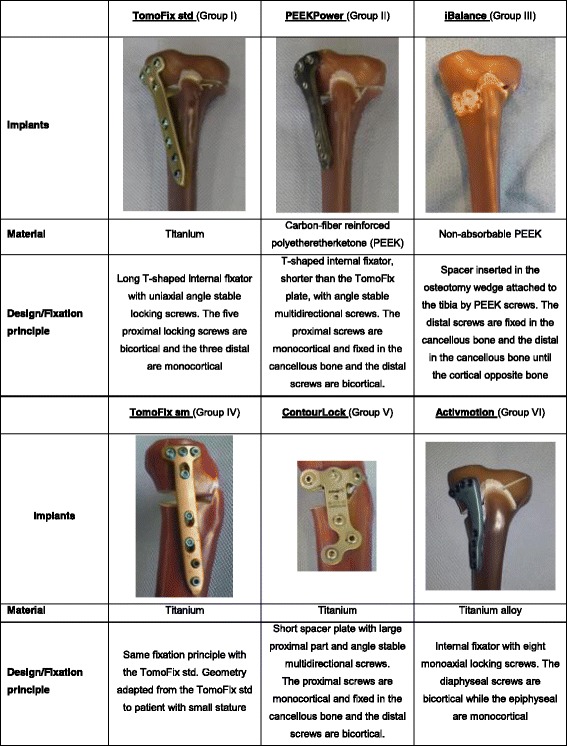
Different HTO implants considered in the present study. The plates are precontoured to fit the proximal tibia head. The ContourLock is wider than the other plates

## Methods

Six large-size fourth generation composite tibia bone models (Sawbones, Pacific Research Laboratories, Inc., Vashon, WA) were used for the tests. Opening wedge proximal medial osteotomies were performed on each of the composite bones in the same way, according to the biplanar technique, by an experienced surgeon. The surgeon fixed the implants according to standard techniques of each implant. The same standardised procedure, as used in the previously performed osteotomy tests (Diffo Kaze, 2016; Diffo Kaze et al., 2015; Maas et al., 2013) was used to prepare the specimens. The six specimens were subdivided according to the tests performed and were associated to the previously tested implants as indicated in Table 2.

**Table 2:** Specimen grouping and assignment, depending on used implants and the performed test

Performed test	Group I; n = 5 Specimens	Group II; *n* = 5 Specimens	Group III; *n* = 6 Specimens	Group IV; n = 5 Specimens	Group V; n = 5 Specimens	Group VI; n = 6 Specimens
Static: single loading to failure test	TomoFix 1	PEEKPower 1	iBalance 1	TomoFix sm 1	Contour Lock 1	Activmotion 1
	TomoFix 2	PEEKPower 2	iBalance 2	TomoFix sm 2	Contour Lock 2	Activmotion 2
Dynamic: cyclic fatigue to failure test	TomoFix 3	PEEKPower 3	iBalance 3	TomoFix sm 3	Contour Lock 3	Activmotion 3
	TomoFix 4	PEEKPower 4	iBalance 4	TomoFix sm 4	Contour Lock 4	Activmotion 4
	TomoFix 5	PEEKPower 5	iBalance 5	TomoFix sm 5	Contour Lock 5	Activmotion 5
			iBalance 6			Activmotion 6

For the static tests, the specimens were subjected to a quasi-static compression displacement-controlled single loading to failure at a speed of 0.1 mm/s. A testing procedure similar to the standardised testing protocol for hip joints (ISO 7206-4, 1989; ISO 7206-6, 1992; ISO 7206-8, 1995) was applied to the dynamic tests of the implant. This consisted of load-controlled cyclical fatigue testing, with stepwise compression sinusoidal (frequency = 5 Hz) loading, where the force amplitude of each step was kept constant with feedback control of the force signal within the hydraulic machine. The lower compressive force limit of each load step was kept constant at 160 N. Starting at 800 N for the first step, the upper compressive force limit was increased stepwise by 160 N after *N* = 20,000 cycles if no failure occurred.

**Fig. 1:**
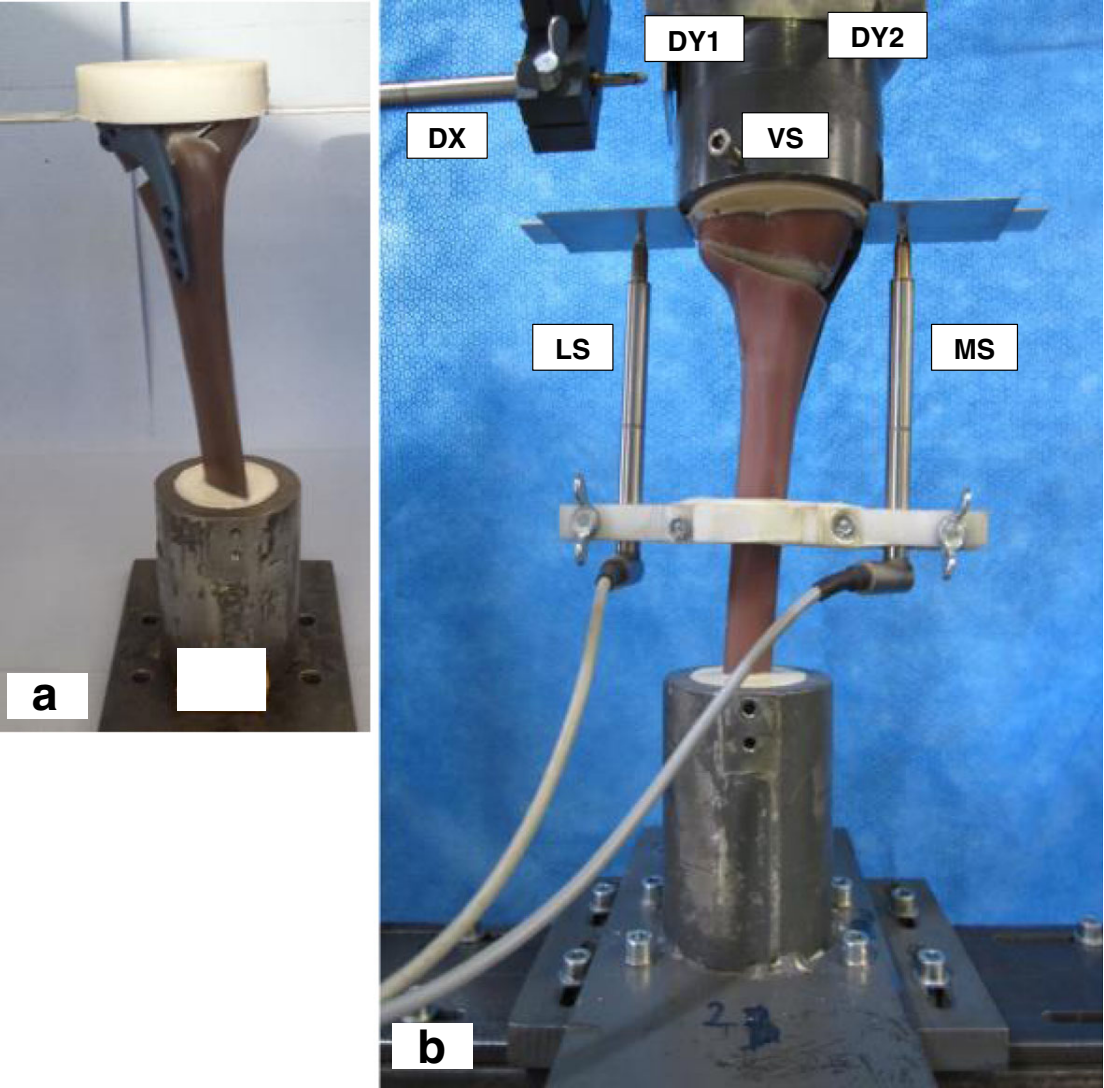
Specimen and sensors’ locations: (**a**) Specimen before mounting to hydraulic press. **b** Specimen under test: The lateral and the medial sensor (LS and MS) register the relative lateral and medial vertical displacements from the tibial head, while VS measured its vertical displacement. The sensors DX, DY1 and DY2 register the horizontal displacements of the tibial head; along the transverse axis for the first and the sagittal axis for the latter

Vertical loading was applied to the tibia head of the specimens (Fig. 1a) through a freely movable support, which allowed any horizontal motion in the transversal plane using three freely rolling metal balls. The Fig. 1b shows the positions of the displacements sensors used to capture the deformation of the specimens. The displacement in the frontal plane on the medial side of the tibia head was measured by the medial sensor MS. A second sensor LS on the lateral side measured the lateral displacement. Three displacement sensors DX, DY1, and DY2 were attached on the freely sliding support in order to measure the horizontal displacements of the tibia head in two perpendicular directions. A fifth displacement sensor VS embedded in the INSTRON machine measured the vertical displacement of piston.

### Failure criteria

**Fig. 2:**
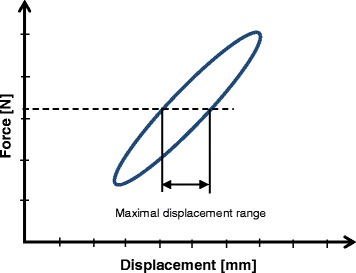
Maximal displacement range within a hysteresis loop of an ideal spring-damper element: The hysteresis loop of an ideal spring-damper element is an inclined ellipsis

The Table 3 summarises the failure criteria that have been considered within the present study. These criteria were already used by Pape et al. (Pape et al., 2010) and were considered in our previous comparative studies. The failure type 3 made it possible to quantify the wobble degree, or stability, of the sample during the cyclic testing. This criterion is checked by plotting the applied sinusoidal force versus the measured displacement of interest, then by measuring the displacement range within the curve obtained (Diffo Kaze, 2016; Diffo Kaze et al., 2015; Maas et al., 2013). If a sinusoidal force is applied on an ideal spring-damper element then the response will be a phase-shifted sinusoidal displacement. Plotting the force versus the displacement leads to an inclined ellipsis, where the maximal displacement, measured as indicated in Fig. 2, is an important characteristic for assessment of stability of the construct.

**Table 3:** Used failure types and their defining criteria (Diffo Kaze, 2016; Diffo Kaze et al., 2015; Maas et al., 2013)

Failure type	Criteria
1	Medial or lateral displacements of the tibial head in relation to the tibial shaft of more than 2 mm equivalent to a rotation of more than 1.4 °. A counter-clockwise rotation corresponds to a valgus malrotation of the tibia head. This criterion can only be checked in the unloaded condition.
2	Visible collapse of lateral cortex. Small hairline cracks are not considered as failure.
3	Maximal displacement range of more than 0.5 mm within one hysteresis loop in the case of cyclic testing only.
4	Cracks of the screws of more than 1 mm

### Permanent deformation and deflection due to plastic deformation during the cyclic testing

The permanent deformation, after unloading the specimen, results from plastic deformation and was estimated as the irrecoverable displacement from the start of the tests at the minimal force of 160 N, considered as nearly zero force. The permanent deflection angle *α*_*p*_ after the collapse of the contralateral cortex during the cyclic tests was determined using the method indicated by Diffo Kaze et al. (Diffo Kaze, 2016; Diffo Kaze et al., 2015; Maas et al., 2013). The deflection angle corresponds to a rotation of the tibia head relative to the shaft. This rotation occurs in the frontal plane, which is the result of a deflection due to the absolute difference between the lateral and the medial displacements. A permanent deflection angle *α*_*p*_greater than 0.024 rad, or 1.4°, corresponds to an occurrence of failure type 1 (Table 3) (Diffo Kaze, 2016; Diffo Kaze et al., 2015; Maas et al., 2013).

### Stiffness of the specimens

The dynamic stiffness of the specimens was determined as a damage indicator during the cyclic tests. It was calculated as the ratio of peak to peak force ∆F to the measured peak to peak displacement ∆X in the same period.

$$ \mathrm{K}=\frac{\Delta  F}{\Delta  X}\kern0.5em . $$

The static stiffness, at the critical state when the damage of the specimen occurs, was calculated as the ratio of the corresponding damage load (F_Damage_) to the corresponding displacement (X_Damage_).

$$ \mathrm{K}=\frac{{\mathrm{F}}_{\mathrm{Damage}}}{{\mathrm{X}}_{\mathrm{Damage}}}. $$

### Statistical analysis

The number of specimens was limited due to financial reasons. There were neither final loads, displacements of the tibia head, nor number of cycles prior to failure that were predefined as reference quantities in the present study. Hence no statistical analyses were performed within the Activmotion groups. Statistical analysis was performed using Microsoft Excel 2010 software (Microsoft Corporation, Redmond, Washington, USA).The t-test for two independent samples was used to compare the ultimate loads, the displacements of the tibia head, the valgus malrotation, the lateral stiffness and the number of cycles prior to failure between the Activmotion group and the others. All statistical tests performed were two-tailed. Statistical significance was considered at *p* < 0.05.

## Results

The same materials and methods from our previously performed and published studies have been used for the specimens with the Activmotion plate (Group VI, Table 2) of the present study. Hence, the results obtained from all these studies are comparable. The published results of our previous studies (Groups I to V, Table 2) are also presented here for comparison purposes.

### Static loading to failure

**Fig. 3:**
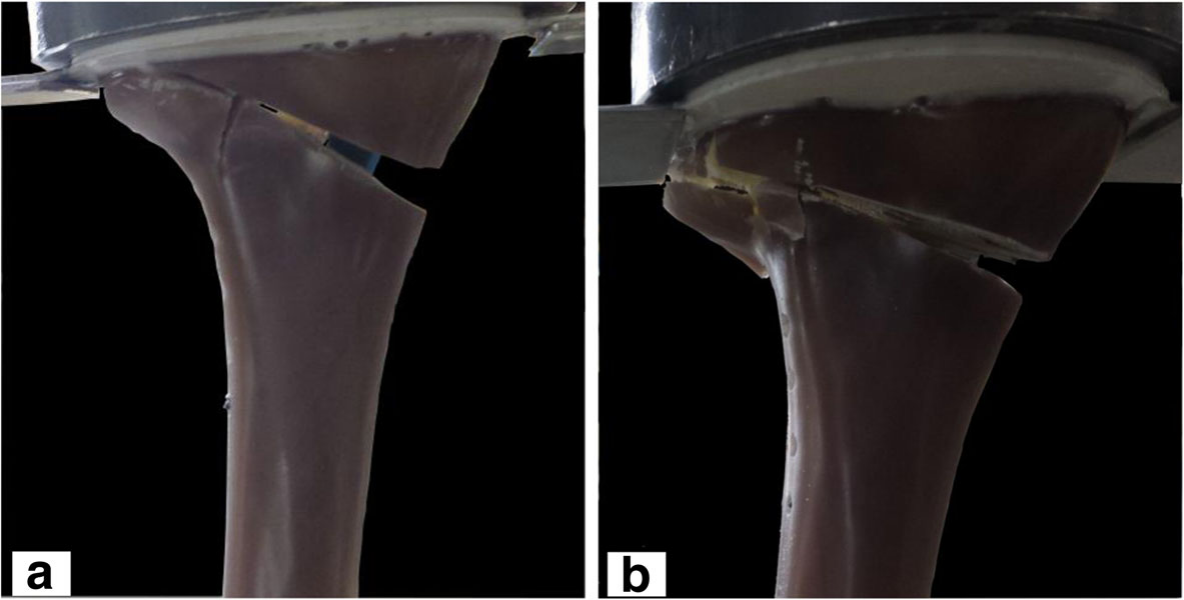
Fracture of the lateral cortical bone in specimens: (**a**) Activmotion 1 and (**b**) Activmotion 2. The opposite cortex was the weak point of the specimens

The specimens Activmotion 1 and 2 failed by fracture of the contralateral cortical bone (Fig. 3). The fracture of the lateral cortex of the specimen Activmotion 1 was abrupt with no observable crack formations prior to the failure.

**Fig. 4:**
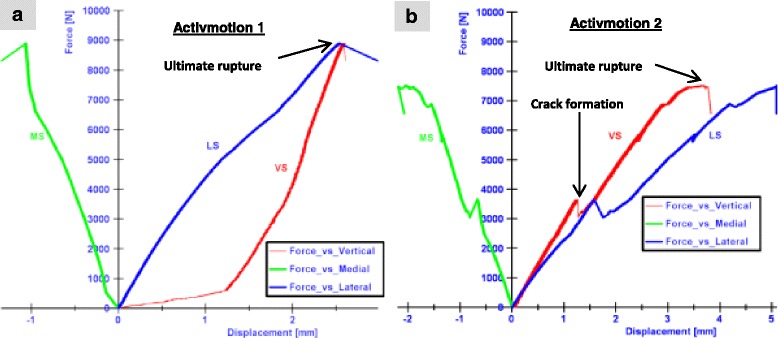
Static test results: (**a**) Activmotion 1: the rupture of the lateral cortex occurred without observable cracks formation. **b** Activmotion 2: Cracks formations preceded the final rupture of lateral cortex

The ultimate fracture in the case of Activmotion 2 was preceded by crack formations as indicated on Fig. 4b, which depicts the characteristic curves (force versus registered displacements) for specimens Activmotion 1 and 2 obtained from the static tests. No defects of the plates or screws were observed.

By considering the direction of the applied load as positive (in the descending vertical direction), the medial displacements (MS) were negative and the lateral displacements (LS) were positive, and greater in magnitude (Fig. 4), hence the tibia plateau of the specimens Activmotion 1 and 2, exhibited a valgus-malrotation in the frontal plane during the static loading.

The results of the static tests performed on the specimens with the Activmotion plates (Group VI, Table 2) are summarised in Table 4. The ultimate load, at which the specimens collapsed during the static tests, was on average 8.2 kN. The lateral displacement at collapse time was on average 3.8 mm. A valgus-malrotation of 1.4° was obtained for the Activmotion 2, which corresponded to a failure type 1. Table 5 gives *p* values obtained from the mean comparison between the Activmotion group and the other groups. The mean and standard deviations were retrieved from our previous studies. All the differences observed were not statistically significant as all p values were greater than 0.05. Comparing Tables 4 and 5 it appears that: (1) the specimens Contour Lock 1 and 2 showed the largest average lateral displacement (4.1 mm) at fracture of the lateral cortex; (2) the iBalance group showed the highest lateral stiffness at ultimate load (3.1 kN/mm); (3) the highest ultimate load was obtained in the Activmotion group; (4) the determined valgus-malrotation of the tibial head was greater, or equal to, the fixed limit of 1.4° of the permanent deflection angle for all implants, except for the iBalance and Activmotion specimens, which showed mean values of 0.9 ° and 1° respectively; and (5) the TomoFix std. group showed the maximal valgus-malrotation at collapse time of the contralateral cortex (2.8°) and the iBalance group showed the minimal (0.9°).

**Table 4:** Static tests summary: Displacements, valgus-malrotation of the tibial head and their corresponding crack and ultimate loads, including mean values and standard deviations (SD)

Specimen	Crack / Ultimate load [kN]	Medial displ. at crack/ ultimate load [mm]	Lateral displ. at crack/ ultimate load [mm]	valgus-malrotation at crack/ ultimate load (°)	Lateral stiffness at crack/ ultimate load [kN/mm]	Failure types
Activmotion 1	- / 8.9	- / 1.3	- / 2.5	- / 0.6	- / 3.6	2
Activmotion 2	3.7 / 7.5	0.7 / 2.1	2.6 / 5.1	0.9 / 1.4	1.4 / 1.5	1 and 2
Mean:	- / 8.2	- / 1.7	- / 3.8	- / 1.0	- / 2.6	
SD ±:	- / 1.0	- / 0.4	- / 1.3	- / 0.4	- / 1.1

**Table 5:** Results of the t-test comparing the previous tested implants to the Activmotion implant. Mean values were compared. All p values were greater than 0.05

Groups		Ultimate load [kN]	Medial displ. at ultimate load [mm]	Lateral displ. at ultimate load [mm]	valgus-malrotation at ultimate load (°)	Lateral stiffness at ultimate load [kN/mm]
TomoFix std.	Mean:	5.3	1.2	4.7	2.8	1.1
	SD ±:	0.1	0.1	0.4	0.2	0.1
	*p* value:	> 0.05	> 0.05	> 0.05	> 0.05	> 0.05
PEEKPower	Mean:	4.4	0.3	3.1	1.6	1.4
	SD ±:	0.1	0.3	0.3	0.1	0.1
	*p* value:	> 0.05	> 0.05	> 0.05	> 0.05	> 0.05
iBalance	Mean:	5.5	0.3	1.9	0.9	3.1
	SD ±:	0.2	0	0.4	0.4	0.7
	*p* value:	> 0.05	> 0.05	> 0.05	> 0.05	> 0.05
TomoFix sm	Mean:	3.4	0.8	2.1	1.4	1.7
	SD ±:	0.3	0.2	0.4	0.1	0.1
	*p* value:	> 0.05	> 0.05	> 0.05	> 0.05	> 0.05
Contour Lock	Mean:	3.6	0.5	4.1	2.2	0.9
	SD ±:	0.5	0	0.2	0.1	0.1
	*p* value:	> 0.05	> 0.05	> 0.05	> 0.05	> 0.05

The overall observation from the static tests is that the Activmotion plate showed higher strength values with smaller deformations when compared to the other implants.

### Fatigue loading to failure

**Fig. 5:**
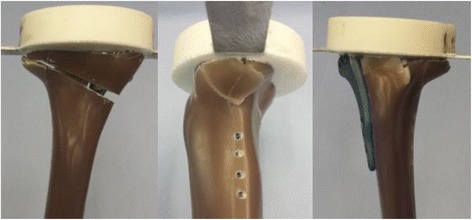
Fracture of the contralateral cortical bone during the fatigue testing: The specimens failed by fracture of the contralateral cortical bone, similar to the static tests

**Fig. 6:**
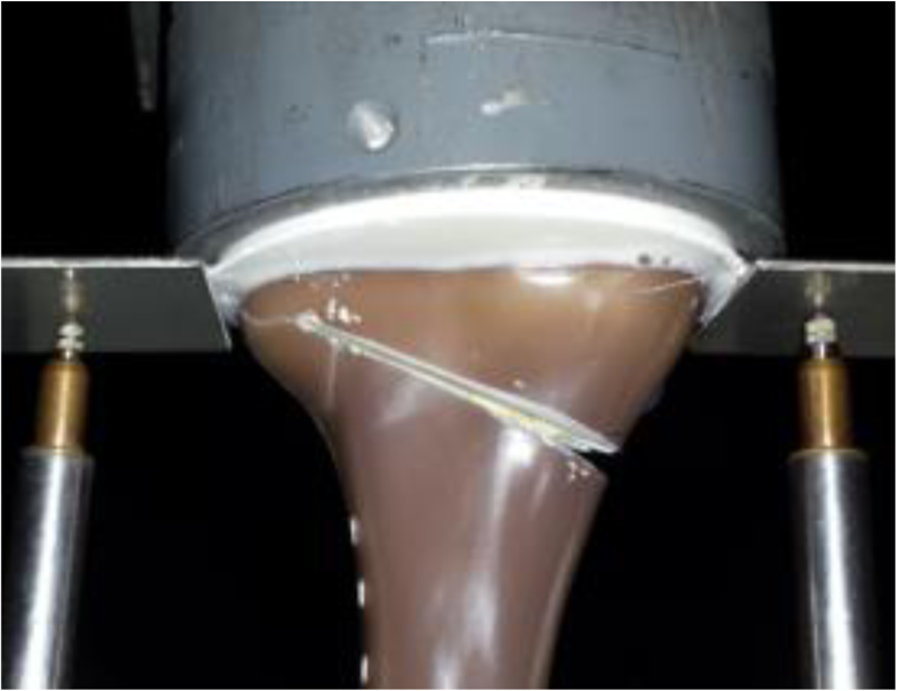
Cracking of the contralateral cortex during the cyclical testing: Unlike the case of Activmotion 4 showed on the picture, the cracking was generally not observable

The fracture of the specimens subjected to cyclical tests occurred in the region of the contralateral cortex (Fig. 5), similar to the static tests. If cracks occurred prior to the final failure of the specimens, they were generally not observable, except in the case of the specimen Activmotion 4 (Fig. 6), where the crack formation was visible. The plates and screws remained undamaged during the cyclical fatigue testing.

**Fig. 7:**
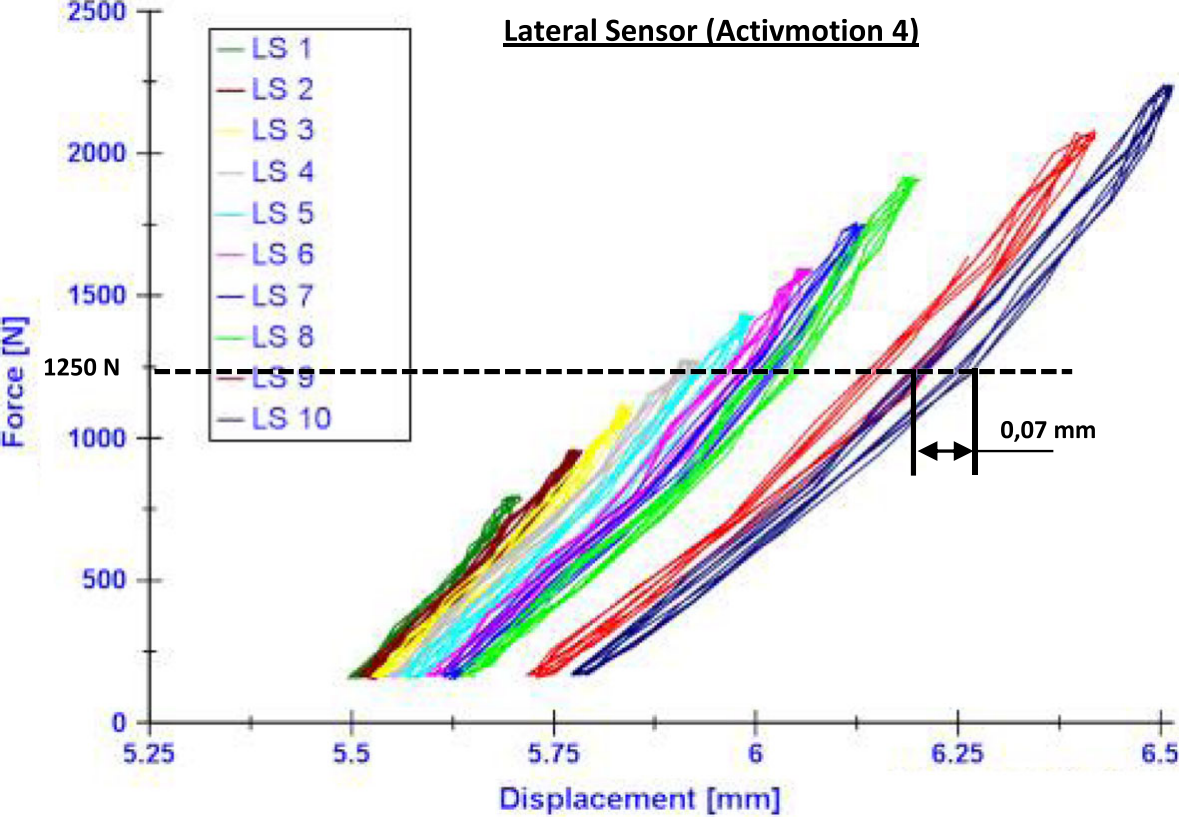
Examples of hysteresis loops (Activmotion 4): Curves force versus lateral displacement. The maximal displacement range, which increase with the failure, is 0,07 mm

The failure type 3, which is checked by means of the maximal displacement range within hysteresis loops, did not occur in the Activmotion group because all the values of the determined maximal range were smaller than 0.5 mm, as shown as an example in Fig. 7. This failure type was only observed in the groups of TomoFix sm and Contour Lock in our previous studies (Diffo Kaze, 2016; Diffo Kaze et al., 2015; Maas et al., 2013), which means there was no failure type 3 for groups I, II, III and IV in the present comparative study.

**Fig. 8:**
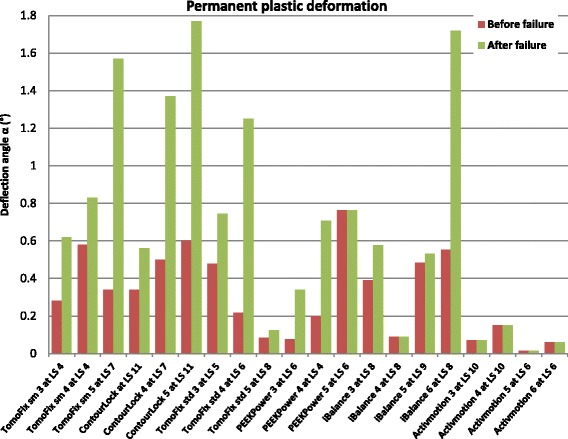
Comparison of the deflection angle or valgus-malrotation of the tibial head before and after the failure for groups 1, 2, 3 and 6: The failure type 1 was observed in the case of the specimen iBalance 6 after the collapse of the opposite cortex. LS “n” means the failure occurred at load step “n”. The values of the first 3 groups are retrieved from our previous studies

During the cyclic loading, the tibia head of all the specimens rotated counter-clockwise, such that the displacement registered by the medial sensor was counted as negative. This was because the descending vertical direction was considered to be positive. The crack formation observed prior to the collapse of the specimen Activmotion 4 (Fig. 7) was not considered as a failure and the other fractures observed were not preceded with visible cracking. Hence, the permanent valgus-malrotations of the tibia before and after the failure were considered to be the same for the group Activmotion. The Fig. 8 recapitulates, for comparison purposes, the permanent deflection angles obtained for all six groups. The load history is indicated with the Load Step number (LSn) at which the failure occurred. No value of the permanent deflection angle was higher than 1.4° in the Activmotion group. The maximal permanent deflection angle was 0.15° in the Activmotion group. This means that the failure type 1, which is characterised by a permanent deflection angle greater than 1.4 °, did not occur for the specimens of group VI subjected to the cyclical loading. The Activmotion group showed smaller permanent deflection angles when compared to the other five groups. The failure type 1 occurred only in the iBalance, TomoFix sm and Countour Lock groups.

The results of fatigue loading to failure of the specimens in groups I to V (Table 2) are from our previous studies, which have been presented here for the sake of comparison together with the results obtained from the testing on the Activmotion (Group VI, Table 2) plate in the Table 6. This table summarises the results of the cyclic fatigue to failure tests by listing the maximal compressive force, lateral and vertical stiffness of the specimens at the beginning of the first load step, the number of cycles performed prior to the failure, and the types of failure. The values reported for the stiffness are those right at the beginning of the first loading step. For group VI, no damage of the fixation system, i.e. failure type 4, was observed; only the failure type 2, i.e. collapse of the contralateral cortex, was observed. Damage of the fixation system occurred in the iBalance group.

**Table 6:** Summary of fatigue failure tests: maximal load, vertical & lateral stiffness, number of cycles (all values prior to failure) and failure types. The values of the first 5 groups have been retrieved from our previous studies and reported here for comparison purposes. All the differences were not statistically significant

Specimen	Maximal load [N]	Vertical stiffness K_V_ [N/mm]	Lateral stiffness K_L_ [N/mm]	Number of cycles	Failure types
TomoFix std. 3	1280	1350	2000	> 60000	2
TomoFix std. 4	1440	2000	2500	> 80000	2
TomoFix std. 5	1760	2500	2200	> 120000	2
Mean:	1.5	1950	2233	> 86000	
SD ±:	0.2	577	252	30550	
PEEKPower 3	1440	2000	2500	> 80000	2
PEEKPower 4	1280	1950	2140	> 60000	2
PEEKPower 5	1440	2785	2250	> 80000	2
Mean:	1.4	2245	2297	> 73000	
SD ±:	0.1	468	184	11500	
iBalance 3	1760	4000	3600	> 120000	2,4
iBalance 4	1760	3000	3400	> 120000	2
iBalance 5	1920	3000	2952	> 140000	2
iBalance 6	1760	3500	2500	> 120000	1,2
Mean:	1.8	3375	3113	125000	
SD ±:	0.1	479	490	10000	
TomoFix sm 3	1280	2200	2000	> 60000	2,3
TomoFix sm 4	1280	1750	1500	> 60000	2,3
TomoFix sm 5	1760	2000	2300	> 120000	1,2
Mean:	1.4	1983	1933	> 80000	
SD ±:	0.3	184	330	28,300	
Contour Lock 3	2400	2100	4400	> 200000	2
Contour Lock 4	1760	2300	2400	> 120000	2
Contour Lock 5	2400	2700	2600	> 200000	1,2,3
Mean:	2.2	2367	3133	173000	
SD ±:	0.4	250	900	37700	
Activmotion 3	2240	2500	6300	> 180000	2
Activmotion 4	2240	2500	2900	> 180000	2
Activmotion 5	1600	2500	4750	> 100000	2
Activmotion 6	1600	3100	5100	> 100000	2
Mean:	1.9	2650	4763	140000	
SD ±:	0.3	260	1219	40000	

Regarding the parameters investigated for the fatigue loading to failure tests, the Contour Lock group showed the highest values, followed by the Activmotion. The highest lateral and medial stiffness was showed by the Activmotion and the iBalance groups respectively. The PEEKPower group showed higher stiffness compared to the TomoFix plates. All *p* values, which resulted from the comparison of the mean values of the Activmotion group to those of the other five groups, were greater than 0.05. Hence, the differences observed were not statistically significant.

**Fig. 9:**
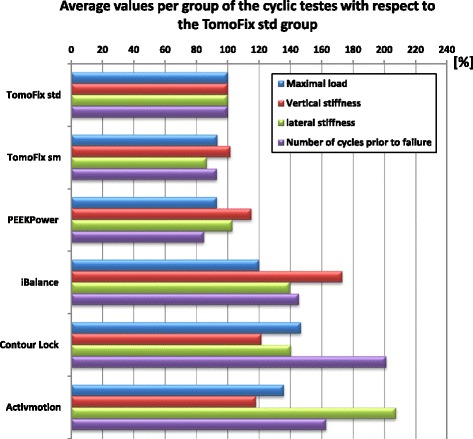
Average relative strength values: The TomoFix std. group has been taken as reference

Figure 9 shows the average relative values per group of the cyclic tests that have been calculated based on Table 6 and by taking the group TomoFix std. as reference.

The life span of the Activmotion specimens prior to failure was, on average, 1.7 higher than that of the TomoFix standard group. The lateral stiffness of the Activmotion group was more than twice that of the TomoFix std. group. The Contour Lock plates showed higher maximal load and number of cycles prior to failure than the Activmotion plates.

## Discussion

In this study the static and the fatigue strength of the size 2 Activmotion plate was investigated and compared with our previous studies using the same experimental setting and protocol. The following five medial open wedge HTO-plates were matched against the Activmotion plate: The TomoFix std. plate, the PEEKPower plate, the iBalance implant, the Contour Lock HTO plate and the TomoFix sm plate. The key findings of the present study were that: (1) the bone-implant construct with the highest stiffness was found to be the size 2 Activmotion plate followed by the Contour Lock plate. (2) The Contour Lock plate provided highest fatigue strength followed by the size 2 Activmotion plate. (3) Static loading tests revealed superior strength of the Activmotion plate followed by the iBalance implant, the TomoFix std., the PEEKPower plate, the Contour Lock and the TomoFix sm plates. (4) All implants withstood the maximal physiological vertical tibiofemoral contact force during slow walking. This force is about 3 times body weight (Heinlein et al., 2009; Taylor et al., 2004), e.g. 2400 N for a patient weighing 80 kg. (5) All tested Activmotion specimens failed during static and cyclic failure tests due to fracture of the lateral cortex, in the same manner as the other five previously tested implants.

Compared to other biomechanical studies (Agneskirchner et al., 2006; Diffo Kaze et al., 2015; Maas et al., 2013; Spahn & Wittig, 2002; Stoffel et al., 2004; Watanabe et al., 2014; Han et al., 2014) that reported fracture of the lateral cortex, the finding (5) of the present study indicates that the lateral cortex is the weakest point of the open wedge HTO. Beside smokers, patients with a fracture of the lateral cortex after HTO exhibit delayed union (Schröter et al., 2015; Takeuchi et al., 2012). This highlights the importance of using an implant that will avoid fracture of the cortical lateral hinge prior to the beginning of gap healing, which takes approximately 3 to 8 weeks (Marsell & Einhorn, 2011). In the cyclic loading fatigue test, the highest strength, i.e. the highest number of cycles prior to failure of the lateral cortex, was found in the Contour Lock group followed by the Activmotion group (Fig. 9). Considering the fact that a healthy active person performs 1 million loading cycles of their limb per year (Baleani et al., 2003; Bergmann et al., 2001; Thielen, 2009), all the plates, except the PEEKPower, would preserve a safe lateral cortex for at least 4 weeks, corresponding to about 80,000 cycles (Table 6), bearing in mind that soft callus formation starts about 2 weeks after fracture (Keita & Perren, 2017).

In the present study, valgus-malrotation relative to the shaft was observed in the frontal plane of the tibia head. This resulted from the difference in magnitude, and the opposite directions of the lateral and the medial displacement. The valgus-malrotation will alter the localisation of the mechanical axis and the primary performed correction. Medial or lateral displacement of the tibial head in relation to the tibial shaft of more than 2 mm was set as failure type 1. This correlated to a limit angular value of 1.4° for the permanent valgus-malrotation due to plastification of the specimen during the cyclic loading of the fatigue to failure tests. The permanent valgus-malrotation was smaller than 1.4° in group VI, which consisted of the Activmotion specimens (Fig. 8), while we previously reported (Diffo Kaze, 2016; Diffo Kaze et al., 2015; Maas et al., 2013) values greater than 1.4° for the specimens iBalance 6, TomoFix sm 4 and ContourLock 5 of group III, group IV and group VI respectively. Hence, it can be assumed that the TomoFix std., the PEEKPower and the Activmotion plates better conserve correction, while the latter performs best for this parameter.

During the static loading to failure test, the average ultimate force of the Activmotion prior to fracture of the lateral cortical hinge was 8.2 kN, a value which is higher compared to the average values from our previous studies, namely 5.5 kN, 5.3 kN, 4.4 kN, 3.6 kN and 3.4 kN for the iBalance, the TomoFix std., the PEEKPower, the Contour Lock and the TomoFix sm group respectively. Hence, the size 2 Activmotion was superior regarding the static force. The iBalance implant showed the smallest mean lateral displacement (1.9 mm) compared to the Activmotion plates, with a lateral displacement of 3,8 mm.

No failure type 3 was observed in the specimens of the Activmotion group. This suggests that the size 2 Activmotion plate offered good stability to the bone-implant construct as the failure type 3 quantifies the wobble degree of the bone-implant construct. The data from our previous studies indicated no failure type 3 in the groups I, II and III, except for twice within the TomoFix sm group and once in the Contour Lock group (Diffo Kaze, 2016; Diffo Kaze et al., 2015; Maas et al., 2013).

Stiffness has been introduced into this study as an additional damage indicator; furthermore, a high stiffness of the lateral side of the bone-implant construct suggests a stable lateral cortical hinge. The size 2 Activmotion plate showed the highest lateral stiffness (4763 N/mm) compared to the other implants. It is important to highlight at this level the anterior medial positioning of the Activmotion plate, which is different to the positioning of all the other implants investigated in the present study, which are all centred on the medial side of the proximal tibia. Blecha et al. (Blecha et al., 2005) investigated the plate positioning by means of finite element method and reported that MOWHTO with medial plate position supports smaller loading than MOWHTO with anteromedial plate position.

We concluded in our previous studies (Diffo Kaze, 2016; Diffo Kaze et al., 2015) that mechanical static and fatigue strength increase with a wider proximal T-shaped plate design together with diverging proximal screws as used in the ContourLock plate, or in a closed-wedge construction as in the iBalance design. Clinical studies, which compared the TomoFix plate to plates that were not included in the present study, reported better clinical results for the TomoFix in terms of implant-related complications, non-unions and stability (Cotic et al., 2015; Jung et al., 2013; Kyung et al., 2015; Saeed & Rae, 2009; Valkering et al., 2009). Furthermore, the PEEKPower plate appeared to be inferior to the TomoFix plates, but (Cotic et al., 2015) reported after a clinical study that the 2nd generation PEEKPower plate is a viable fixation device for open-wedge HTO with osteotomy gaps up to 12 mm. This brings up the issue of the correlation between mechanical strength and better clinical outcomes. Nevertheless, all fixation devices should provide sufficient stability to the tibia until bony union. We did not find clinical studies between the TomoFix, the ContourLock, the iBalance and the size 2 Activmotion implants, as the size 2 Activmotion plate was recently introduced to the market.

Since mechanical stimulation can induce fracture healing or alter its biological pathway (Claes et al., 1997; Claes et al., 1998; Goodship & Kenwright, 1985; Isaksson, 2012), the clinical performance of implants could not be only correlated to their mechanical performance in terms of static and fatigue strength. But it is a necessary condition to have a minimum stability for the functionality of the bone implant-constructs. Of course there are other aspects that have to be taken into account. Although not investigated here, it is important to mention the effects that implants have on callus formation and bone healing, often referred to as “callus massage”, which is explained by the strain theory (Hente et al., 1993; Hente et al., 2001; Nelissen et al., 2010; Perren, 2002; Perren, 2010; Schröter et al., 2011; Staubli & Jacob, 2010). As the effect of the flexibility of the bone-implant construct on the callus massage was not so far investigated in the present study, we cannot draw conclusions regarding this aspect.

Limitations of this study could be the limited number of specimens per group and the fact that bone healing normally takes place a few days postoperatively, before high loading cycle numbers are reached. Hence one should proceed cautiously when transferring the present results to clinical settings.

## Conclusions

Overall, regarding all of the analysed strength parameters, the size 2 Activmotion plate provided equivalent or higher mechanical stability compared to the previously tested implants. Implants with a metaphyseal slope adapted to the tibia anatomy, and positioned more anteriorly on the proximal medial side of the tibia, should provide good mechanical stability.

## Abbreviations

HTO: High tibial osteotomy
PEEK: Polyetheretherketon
SD: Standard deviation
sm: small stature
std.: standard
